# Concomitant valve surgery is associated with worse outcomes in surgical treatments of post-infarction ventricular aneurysm

**DOI:** 10.3389/fcvm.2023.1194374

**Published:** 2023-08-15

**Authors:** Yuqi Liu, Ziwen Cai, Li Xu, Yidan Zheng, Ming Chen, Nianguo Dong, Si Chen

**Affiliations:** Department of Cardiovascular Surgery, Union Hospital, Tongji Medical College, Huazhong University of Science and Technology, Wuhan, China

**Keywords:** ventricular aneurysm, valve surgery, long-term follow-up, coronary artery bypass graft, ventricular reconstruction

## Abstract

**Objective:**

To evaluate the impact of concomitant valve surgery on the prognosis of patients who experienced coronary artery bypass graft (CABG) with/without ventricular reconstruction for the ventricular aneurysm.

**Methods:**

In our department, 354 patients underwent CABG with/without ventricular reconstruction for a ventricular aneurysm from July 23rd, 2000 to December 23rd, 2022. A total of 77 patients received concomitant valve surgery, 37 of whom underwent replacement, and 40 of whom underwent repair. The baseline characteristics, prognostic, and follow-up information were statically analyzed. Univariate and multivariate Cox regression analyses were applied to identify the risk factors of long-term outcomes.

**Results:**

Compared with patients who did not undergo valvular surgery, patients who experienced concomitant valve surgical treatments had a significantly lower survival rate (*p* = 0.00022) and a longer total mechanical ventilation time. Subgroup analysis indicated that the options of repair or replacement exhibited no statistically significant difference in postoperative mortality (*p* = 0.44) and prognosis. The multivariate Cox regression analysis suggested that the pre-operative cholesterol level (HR = 1.68), postoperative IABP (HR = 6.29), NYHA level (HR = 2.84), and pre-operative triglyceride level (HR = 1.09) were independent and significant predictors for overall all-cause mortality after surgery.

**Conclusion:**

Concomitant valve surgery was considerably related to a higher risk of postoperative mortality in patients with post-infarction ventricle aneurysms who underwent surgical treatments. No significant difference in the prognosis outcomes was observed between the operating methods of repair or replacement valve surgery.

## Introduction

Acute myocardial infarction (AMI) poses a severe threat to human life and health owing to its long-term complications including ischemia, arrhythmia, and mechanical complications, although reperfusion therapies can somewhat reduce the short-term fatalities (1).

Ventricular aneurysm accounts for the most common post-infarction mechanical complication, especially under the condition of large infarcts or delayed reperfusion. The aneurysm results from ventricular remodeling after AMI and fails to perform the normal function of myocardial contraction. Surgical treatments, including coronary artery bypass graft (CABG) and ventricular reconstruction, are suggested to restore the blood perfusion and ventricular geometry for the post-infarction ventricular aneurysm (2, 3).

Valve regurgitation is another frequent post-infarction complication. Post-infarction valve damage may result from papillary muscle ischemia or ventricular remodeling, leading to severe valve regurgitation and seriously destabilizing normal hemodynamics (4, 5). Intractably, the surgical strategies for ischemic mitral valve regurgitation are controversial, and whether the options of replacement or repair have different prognoses when the ventricular aneurysm is presented requires further evidence from further clinical research (1, 6).

In this study, we conducted a retrospective study among 354 patients with ventricular aneurysms after myocardial infarction, investigating the long-term outcomes and associated prognostic factors of surgical treatments with or without valve surgery. Moreover, the long-term survival probability among options of repair or replacement of valve dysfunction combined with the ventricular aneurysm was explored.

## Methods

### Study population

**Figure 1:**
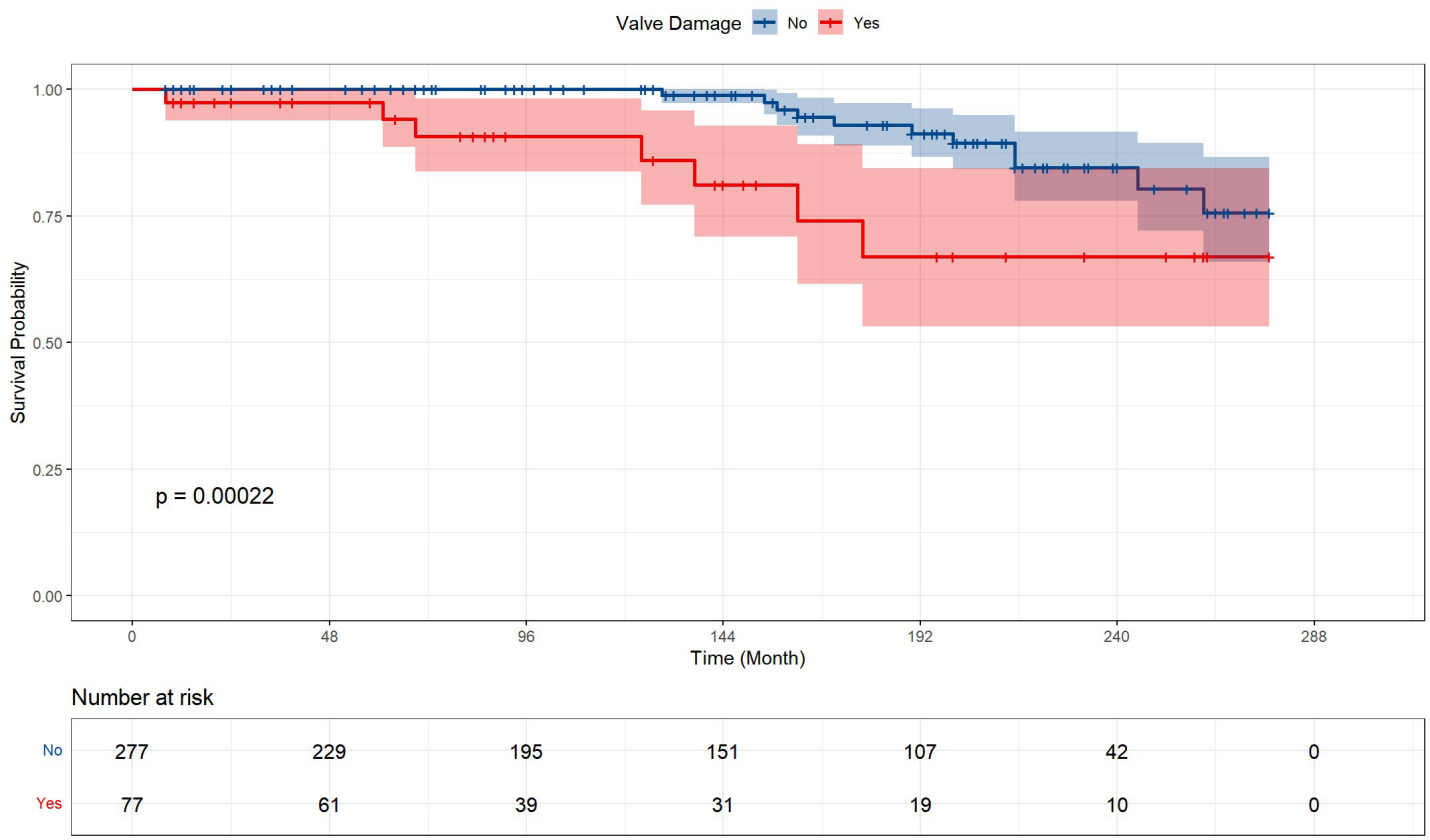
Study cohort design flow chart. A total of 357 patients received surgical treatment for post-infarction aneurysm during the study period. Among them, 190 patients underwent regular surgical intervention including coronary artery bypass graft the infarcted area excising, and ventricular defect closing. 77 patients received valve surgery in addition to standard surgical intervention, of which 37 adopted valve replacement and 40 adopted valve repair.

This retrospective study reviewed data from patients who underwent surgical treatments for ventricular aneurysms after myocardial infarction in our center from July 1, 2000 to December 31, 2022. Patients under 18 years of age or who had excessive data missing were excluded from the study. Patients with acute mitral regurgitation and hemodynamic instability due to papillary muscle rupture from AMI were also not included in the study. Our institution applied an electronic medical records system, from which the baseline biochemical characteristics and clinical measures were conducted (Figure 1).

### Ethical statement

The ethics approval was obtained from the Ethics Committee of Tongji Medical College of Huazhong University of Science and Technology (IORG No: IORG0003571). All patients signed the informed consent in accordance. The study protocol was under the guidance of the national protocol for China category I and conformed to the Declarations of Helsinki and Istanbul.

### Follow-up data and outcome

The clinical follow-up information of recipients was collected from regular clinical visits, telephone interviews, or the Internet. Patients were followed up until September 12, 2022. Overall survival was defined as the absence of all-cause mortality from surgical treatments to the last time following. The primary endpoint was defined as the composite of death due to any cause, including coronary artery disease and cerebrovascular events.

### Data collection

The baseline characteristics contained the pre-operative variables and personal history. The pre-operative variables consisted of demographic data, pre-operative laboratory indicators, and pre-operative treatments. The demographic data included gender, age, and Body Mass Index (BMI). The pre-operative laboratory indicators included total bilirubin (T-Bil), direct bilirubin (D-Bil), aspartate aminotransferase (AST), alanine aminotransferase (ALT), blood urea nitrogen (BUN), serum creatinine (SCr), urea acid (UA), fasting blood glucose, total cholesterol, low-density lipoprotein cholesterol (LDL-C), high-density lipoprotein cholesterol (HDL-C), triglyceride (TG). The personal history included a history of peripheral vascular disease, hyperlipidemia, cerebral infarction, liver disease, chronic kidney disease, surgery, percutaneous coronary intervention, hypertension, continuous renal replacement therapy (CRRT), and diabetes.

Echocardiographic studies were performed with a transmitting frequency from 1.7 to 4.0 MHz on commercially available equipment (Philips EPIQ CVx). The left ventricular ejection fraction (LVEF) was measured by Simpson's method. Cardiac chamber dimensions, including left ventricular (LV) diameter, right ventricular (RV) diameter, left atrial (LA) diameter, right atrial (RA) diameter, interventricular septum (IVS) thickness, ascending aorta inner (AAO) diameter, and pulmonary arterial (PA) inner diameter were measured by dual-mode (2D) echocardiography. The location and size were determined by echocardiography. The left heart enlargement was defined as patients with LV diastolic dimension greater than 54.3 mm for men and 48.6 mm for women according to the guideline (7).

Postoperative events data were also included. Early-postoperative events included postoperative use of CRRT, respiratory, neurological, renal complications, liver damage, septic shock, secondary thoracotomy, and ICU stay time. The definitions of the above complications are described following the definitions in previous studies (8, 9).

### Surgical technique

The ascending aorta was cannulated for the arterial line after a median sternotomy. A single-stage venous cannula was inserted through the right atrial auricle, and aortic root venting was used. Cold crystalloid cardioplegia was administrated through the antegrade route. Cardiopulmonary bypass with moderate systemic hypothermia (30–32°C) and moderate hemodilution (Hct > 0.22) was used. Intermittent cold crystalloid cardioplegia was administrated through the antegrade route in all patients. All patients underwent the CABG procedure, and the internal thoracic artery was applied as the graft. The surgical technique of ventricular reconstruction included three types, namely, linear repair with plication or excision and ventricular reconstruction with endoventricular patches, as described in previous studies. The linear suture method resects the scarred tissue on the LV free wall the incision was closed by direct sutures, and the method using the patch involves incision of the infarct and fixation of a polyester patch to that opening and elimination of the motor or motion-impaired segment (10–12). The repair techniques are adopted according to the lesion location determined by pre-operative echocardiography. Artificial chords are used in the prolapsed posterior leaflet and anterior leaflet. Mitral annuloplasty was performed using a C-ring mitral prosthetics around the posterior leaflet. All procedures were elective.

### Statistical analysis

R version 4.1.2 with the packages Survminer, survival, ggplot2, dplyr, and tableone were used for statistical analysis. Categorical variables were presented as the number (percentage). The continuous variables were presented as mean ± standard deviation (SD) for variables following a normal (Gaussian) distribution. The comparisons among groups were performed by the Chi-square test or Fisher's precision probability test for categorical variables, and the Mann–Whitney *U* rank-sum test and unpaired t test for continuous variables, as appropriate. The univariable survival analysis and the difference between subgroups were statistically evaluated by the Kaplan–Meier method and examined by the log-rank test. Hazard ratios (HR) and corresponding 95% confidence intervals (CI) were estimated by the univariate and multivariate Cox proportional hazards regression model. A two-tailed *p*-value <0.05 was defined as statistical significance.

## Results

A total of 354 patients received surgical treatment for post-infarction aneurysms during the study period. The study included 290 males and 64 females. Among them, 190 patients underwent regular surgical intervention including coronary artery bypass grafting, the infarcted area excising, and ventricular defect closing. Thirty-eight patients received concomitant valve surgery (replacement or repair) in addition to standard surgical intervention, of which 37 adopted replacement and 40 adopted repair.

### Baseline characteristics of patients

The baseline characteristics of different surgical options are separately reported in Table 1. The relationship and differences in clinical characteristics, personal history, and blood biomarkers were analyzed.

**Table 1:** Baseline characteristics of study participants.

	Overall	No valve surgery	Valve surgery	*p*
*N*	354	277	77	
Clinical characteristics				
Gender (%)				0.954
Male	290 (81.9)	227 (81.9)	63 (81.8)	
Female	64 (18.1)	50 (18.1)	14 (18.2)	
Age (year)	57.80 (8.08)	57.67 (8.22)	58.29 (7.60)	0.554
BMI (kg/m^2^)	24.58 (3.38)	24.39 (3.50)	25.27 (2.86)	0.042
Hypertension (%)				<0.001
I	50 (14.1)	32 (11.6)	18 (23.4)	
II	40 (11.3)	24 (8.7)	16 (20.8)	
III	102 (28.8)	79 (28.5)	23 (29.9)	
NYHA level (%)				<0.001
1	64 (18.1)	50 (18.1)	14 (18.2)	
2	130 (36.7)	115 (41.5)	15 (19.5)	
3	124 (35.1)	84 (30.3)	40 (52.0)	
4	36 (10.2)	28 (10.1)	8 (10.4)	
Left Heart Enlargement (%)	240 (67.8)	181 (65.3)	59 (76.6)	0.073
HR (bpm)	77.49 (17.23)	78.08 (17.74)	75.35 (15.14)	0.219
Echocardiography examinations				
AAO (cm)	3.38 (0.36)	3.37 (0.38)	3.41 (0.29)	0.418
LA (cm)	4.13 (0.57)	4.05 (0.52)	4.41 (0.62)	<0.001
LV (cm)	5.79 (0.73)	5.70 (0.72)	6.11 (0.71)	<0.001
IVS (cm)	0.94 (0.21)	0.93 (0.22)	1.00 (0.15)	0.007
RA (cm)	3.75 (0.63)	3.71 (0.66)	3.88 (0.51)	0.037
RV (cm)	3.57 (0.51)	3.53 (0.48)	3.70 (0.58)	0.008
PA (cm)	2.52 (0.38)	2.50 (0.38)	2.60 (0.37)	0.040
LVEF (%)	44.85 (25.01)	44.70 (27.78)	45.41 (10.06)	0.824
LV End-Diastolic Volume (mL)	227.07 (76.98)	224.87 (77.15)	235.10 (76.86)	0.470
LV End-Systolic Volume (mL)	157.08 (64.37)	160.50 (66.41)	144.56 (55.30)	0.177
Mean mitral regurgitation of group (m/s)	0.22 (1.01)	0.25 (1.06)	0.14 (0.81)	0.567
Blood biomarkers				
T Bil (mg/dl)	13.43 (8.19)	13.66 (8.66)	12.60 (6.22)	0.314
D Bil (mg/dl)	4.91 (3.72)	5.05 (3.87)	4.39 (3.05)	0.17
ALT (unit)	30.03 (21.94)	30.46 (23.28)	28.48 (16.29)	0.485
AST (unit)	27.66 (22.40)	28.47 (24.35)	24.74 (12.89)	0.197
BUN (mmol/L)	6.67 (2.97)	6.66 (3.11)	6.70 (2.42)	0.924
SCr (µmol/L)	92.89 (60.58)	92.93 (66.85)	92.77 (28.52)	0.984
UA (µmol/L)	381.65 (124.36)	374.42 (125.79)	407.66 (116.18)	0.038
Total cholesterol (mg/dl)	4.02 (1.84)	3.94 (1.48)	4.31 (2.75)	0.111
Triglycerides (mmol/L)	2.14 (2.77)	2.09 (1.82)	2.33 (4.85)	0.491
HDL-C (mmol/L)	1.40 (1.36)	1.35 (1.14)	1.61 (1.95)	0.14
LDL-C (mmol/L)	3.57 (4.45)	3.43 (3.89)	4.05 (6.06)	0.284
Coronary artery lesion				
LAD (%)	178 (50.3)	140 (50.5)	38 (49.4)	0.898
LCX (%)	168 (47.5)	136 (49.1)	32 (41.6)	0.249
RCA (%)	168 (47.5)	132 (47.7)	36 (46.8)	0.898
PDA (%)	78 (22.0)	58 (20.9)	20 (26.0)	0.354

BMI, body mass index; NYHA, New York heart association; HR, heart rate; AAO, ascending aorta; LA, left atrium; LV, left ventricular; IVS, ventricular septum; RA, right atrium; RV, right ventricular; PA, pulmonary artery; LVEF, left ventricular ejection fraction; T Bil, total bilirubin; D Bil, direct bilirubin; ALT, alanine transaminase; AST, aspartate transaminase; BUN, blood urea nitrogen; SCr, serum creatinine; UA, uric acid; HDL-C, high-density lipoprotein cholesterol; LDL-C, low-density lipoprotein cholesterol; LAD, left anterior descending; LCX, left circumflex artery; RCA, right coronary artery; PDA, posterior descending artery.

Patients who received concomitant valve surgery tended to have a more severe condition of hypertension (*p* = 0.01), and higher BMI (*p* = 0.042). Echocardiography examination demonstrated that patients who required valvular surgery had significantly larger cardiac cavity dimensions, with greater diameters of LA (*p* < 0.001), LV (*p* < 0.001), IVS (*p* = 0.007), RA (*p* = 0.037), RV (*p* = 0.008), and PA (*p* = 0.040). Experimental examinations of blood biomarkers presented no significant difference between the two groups.

### Postoperative events and mortality

**Figure 2:**
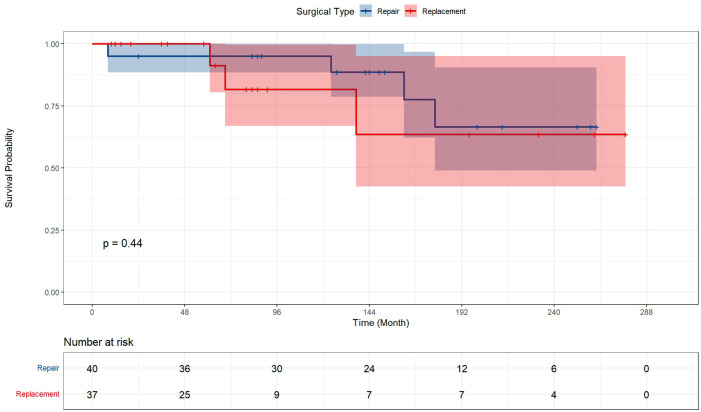
Kaplan–Meier analysis estimates survival after surgery. Kaplan–Meier analysis estimates survival after surgery with log-rank test, comparing the outcomes between ventricular aneurysm patients with or without concomitant valve surgery and receiving corresponding valve surgery.

Table 2 and Figure 2 presented the prognosis outcomes after surgery. The patients receiving valve surgery had significantly lower survival probability (*p* = 0.00022), longer postoperative ICU time (*p* = 0.035), postoperative endotracheal intubation time (*p* = 0.027), and longer total mechanical ventilation time (*p* = 0.004). In addition to longer total mechanical ventilation time, valve surgery cohort patients suffered higher percentages of respiratory complications, renal insufficiency, infection, sputum culture positive, and longer time of ICU stay, endotracheal intubation, intra-aortic balloon pump (IABP), hospital stay after surgery, and all-cause mortality. However, these prevalence differences between groups fail to gain statistical significance.

**Table 2:** Prognostic information for patients undergoing surgery for post-infarction ventricular aneurysm.

	Overall	No valve surgery	Valve surgery	*p*
*n*	354	277	77	
Tracheostomy (%)	14 (4.0)	8 (2.9)	6 (7.8)	0.089
Patients underwent ventricular reconstruction (%)	190 (53.6)	178 (64.3)	12 (15.6)	<0.001
Postopreative IABP (%)	130 (36.7)	98 (35.4)	32 (41.6)	0.350
Respiratory complications (%)	58 (16.4)	40 (14.4)	18 (23.4)	0.080
Renal insufficiency (%)	8 (2.3)	6 (2.2)	2 (2.6)	0.686
Infection (%)	56 (15.8)	40 (14.4)	16 (20.8)	0.216
Sputum culture positive (%)	54 (15.3)	38 (13.7)	16 (20.8)	0.151
rebridge (%)	4 (1.1)	2 (0.7)	2 (2.6)	0.207
ACS (%)	14 (4.0)	8 (2.9)	6 (7.8)	0.089
Postoperative ICU time (hour)	153.95 (114.86)	147.18 (101.50)	178.34 (151.98)	0.035
Postoperative endotracheal intubation time (hour)	60.92 (47.23)	58.00 (44.18)	71.40 (55.97)	0.027
Postoperative IABP time (hour)	58.15 (89.40)	55.88 (87.09)	66.31 (97.41)	0.366
Postoperative hospital stay (day)	19.15 (9.06)	19.04 (9.16)	19.57 (8.71)	0.647
Total mechanical ventilation time (hour)	66.63 (80.60)	60.20 (46.17)	89.77 (147.44)	0.004
One-year LVEF (%)	43.05 (11.34)	43.31 (11.91)	41.44 (7.18)	0.651
All-cause mortality (%)	26 (7.34)	16 (5.78)	10 (13.0)	0.032

IABP, intra-aortic balloon pump; ACS, acute coronary syndrome; ICU, intensive care unit; LVEF, left ventricular ejection fraction.

### Univariate and multivariate Cox analysis

Univariate and multivariate Cox proportional hazards regression models were conducted to identify risk factors for all-cause postoperative mortality. The result of the univariate analysis showed that the risk factors of mortality were total cholesterol (HR = 1.22, 95% CI, 1.08–1.37, *p* < 0.01), LDL-C (HR = 1.08, 95% CI, 1.02–1.14, *p* = 0.01), age (HR = 1.09, 95% CI, 1.02–1.16, *p* = 0.01), triglycerides (HR = 1.09, 95% CI, 1.01–1.17, *p* = 0.02), New York Heart Association (NYHA) level (HR = 1.88, 95% CI, 1.06–3.36, *p* = 0.03), and concomitant valve surgery (HR = 2.93, 95% CI, 1.09–7.83, *p* = 0.03).

Multivariate analysis showed that total cholesterol (HR = 1.68, 95% CI, 1.45–1.94, *p* < 0.001), postoperative IABP (HR = 6.29, 95% CI, 2.05–19.3, *p* = 0.0013), NYHA level (HR = 2.84, 95% CI, 1.50–5.41, *p* = 0.0014), and triglycerides (HR = 1.09, 95% CI, 1.01–1.17, *p* = 0.0283) were independent and significant predictors of worse outcomes after surgery (Table 3).

**Table 3:** Results of univariate and multivariate Cox analysis for the prognosis after surgery.

Univariate Cox analysis	Hazard ratio	*p*
Total cholesterol	1.22 (1.08–1.37)	<0.01
LDL-C	1.08 (1.02–1.14)	0.01
Age	1.09 (1.02–1.16)	0.01
Triglycerides	1.09 (1.01–1.17)	0.02
NYHA level	1.88 (1.06–3.36)	0.03
Valve damage	2.93 (1.09–7.83)	0.03
ALT	1.01 (1.00–1.03)	0.14
Operation history	0.52 (0.17–1.57)	0.24
Hypertension	1.22 (0.87–1.71)	0.25
D-Bil	0.93 (0.78–1.12)	0.45
BMI	0.96 (0.83–1.11)	0.55
HDL-C	1.08 (0.81–1.43)	0.60
BUN	1.03 (0.93–1.14)	0.62
AST	1.00 (0.97–1.02)	0.67
Left heart enlargement	0.89 (0.35–2.27)	0.82
T-Bil	0.99 (0.93–1.06)	0.84
Gender	0.89 (0.26–3.06)	0.85
SCr	1.00 (1.00–1.01)	0.92
HR	1.00 (0.97–1.03)	0.98
Multivariate Cox analysis	Hazard ratio	*p*
Total cholesterol	1.68 (1.45–1.94)	<0.001
Postopreative IABP	6.29 (2.05–19.3)	0.0013
NYHA level	2.84 (1.50–5.41)	0.0014
Triglycerides	1.09 (1.01–1.17)	0.0283
Age	1.02 (0.95–1.09)	0.5623

LDL-C, low-density lipoprotein cholesterol; ALT, alanine transaminase; D Bil, direct bilirubin; BMI, body mass index; HDL-C, high-density lipoprotein cholesterol; BUN, blood urea nitrogen; T Bil, total bilirubin; SCr, serum creatinine; HR, heart rate; IABP, intra-aortic balloon pump; NYHA, New York heart association.

### Subgroup analysis

The clinical characteristics and prognosis information were also evaluated in Table 4. No baseline heterogeneity was determined between patients with the option of repair and replacement except for the interventricular septal wall (*p* < 0.001) and D Bil (*p* = 0.017). The results of the prognosis analysis presented that the options of valve surgery exerted no significant influence on prognostic outcomes, including post-surgery mortality, ICU information, and complications.

**Table 4:** Subgroup analysis of patients receiving concomitant valve surgery.

	Overall	Repair	Replacement	*p*
*n*	77	40	37	
Clinical characteristics				
Gender (%)				
male	63 (81.8)	30 (75.0)	33 (89.2)	0.143
female	14 (18.2)	10 (25.0)	4 (10.8)	
Age (year)	58.29 (7.60)	59.90 (6.97)	56.54 (7.95)	0.052
BMI (kg/m^2^)	25.27 (2.86)	25.33 (2.82)	25.20 (2.94)	0.837
HR (bpm)	75.35 (15.14)	75.85 (13.85)	74.81 (16.60)	0.766
Echocardiography examinations				
AAO (cm)	3.41 (0.29)	3.34 (0.33)	3.48 (0.24)	0.047
LA (cm)	4.41 (0.62)	4.45 (0.56)	4.36 (0.69)	0.528
LV (cm)	6.11 (0.71)	6.24 (0.71)	5.97 (0.68)	0.085
IVS (cm)	1.00 (0.15)	0.93 (0.14)	1.08 (0.12)	<0.001
RA (cm)	3.88 (0.51)	3.86 (0.53)	3.91 (0.49)	0.698
RV (cm)	3.70 (0.58)	3.70 (0.61)	3.70 (0.56)	0.97
PA (cm)	2.60 (0.37)	2.68 (0.36)	2.52 (0.36)	0.053
LVEF (%)	45.41 (10.06)	45.07 (10.56)	45.79 (9.63)	0.757
Blood biomarkers				
T Bil (mg/dl)	12.60 (6.22)	13.39 (6.64)	11.74 (5.69)	0.246
D Bil (mg/dl)	4.39 (3.05)	5.18 (3.78)	3.54 (1.66)	0.017
ALT (unit)	28.48 (16.29)	28.60 (15.29)	28.35 (17.53)	0.947
AST (unit)	24.74 (12.89)	27.90 (15.90)	21.32 (7.33)	0.024
BUN (mmol/L)	6.70 (2.42)	6.71 (1.89)	6.68 (2.92)	0.964
SCr (µmol/L)	92.77 (28.52)	88.09 (20.78)	97.82 (34.62)	0.135
UA (µmol/L)	407.66 (116.18)	421.53 (137.53)	392.66 (86.96)	0.279
Total cholesterol (mg/dl)	4.31 (2.75)	4.41 (3.37)	4.21 (1.91)	0.763
Triglycerides (mmol/L)	2.33 (4.85)	2.98 (6.61)	1.64 (1.16)	0.23
HDL-C (mmol/L)	1.61 (1.95)	1.98 (2.53)	1.21 (0.90)	0.084
LDL-C (mmol/L)	4.05 (6.06)	5.23 (7.76)	2.78 (3.03)	0.076
Prognosis information				
Tracheostomy (%)	6 (7.8)	4 (10.0)	2 (5.4)	0.546
Postopreative IABP (%)	32 (41.6)	20 (50.0)	12 (32.4)	0.165
Respiratory complications (%)	18 (23.4)	6 (15.0)	12 (32.4)	0.106
Renal insufficiency (%)	2 (2.6)	2 (5.0)	0 (0.0)	0.494
Infection (%)	16 (20.8)	6 (15.0)	10 (27.0)	0.263
Sputum culture positive (%)	16 (20.8)	6 (15.0)	10 (27.0)	0.263
Rebridge (%)	2 (2.6)	2 (5.0)	0 (0.0)	0.494
Postoperative ICU time (hour)	177.14 (152.99)	186.80 (184.15)	166.70 (111.63)	0.568
Postoperative endotracheal intubation time (hour)	70.84 (56.45)	76.60 (66.71)	64.61 (42.76)	0.355
Postoperative IABP time (hour)	66.91 (97.14)	66.85 (89.67)	66.97 (105.87)	0.996
Postoperative hospital stay (day)	19.38 (8.98)	20.55 (9.72)	18.11 (8.05)	0.236
Total mechanical ventilation time (hour)	89.61 (147.50)	111.40 (199.28)	66.05 (42.50)	0.179

BMI, body mass index; HR, heart rate; AAO, ascending aorta; LA, left atrium; LV, left ventricular; IVS, ventricular septum; RA, right atrium; RV, right ventricular; PA, pulmonary artery; LVEF, left ventricular ejection fraction; T Bil, total bilirubin; D Bil, direct bilirubin; ALT, alanine transaminase; AST, aspartate transaminase; BUN, blood urea nitrogen; SCr, serum creatinine; UA, uric acid; HDL-C, high-density lipoprotein cholesterol; LDL-C, low-density lipoprotein cholesterol; IABP, intra-aortic balloon pump; ACS, acute coronary syndrome; ICU, intensive care unit; LVEF, left ventricular ejection fraction.

**Figure 3:**
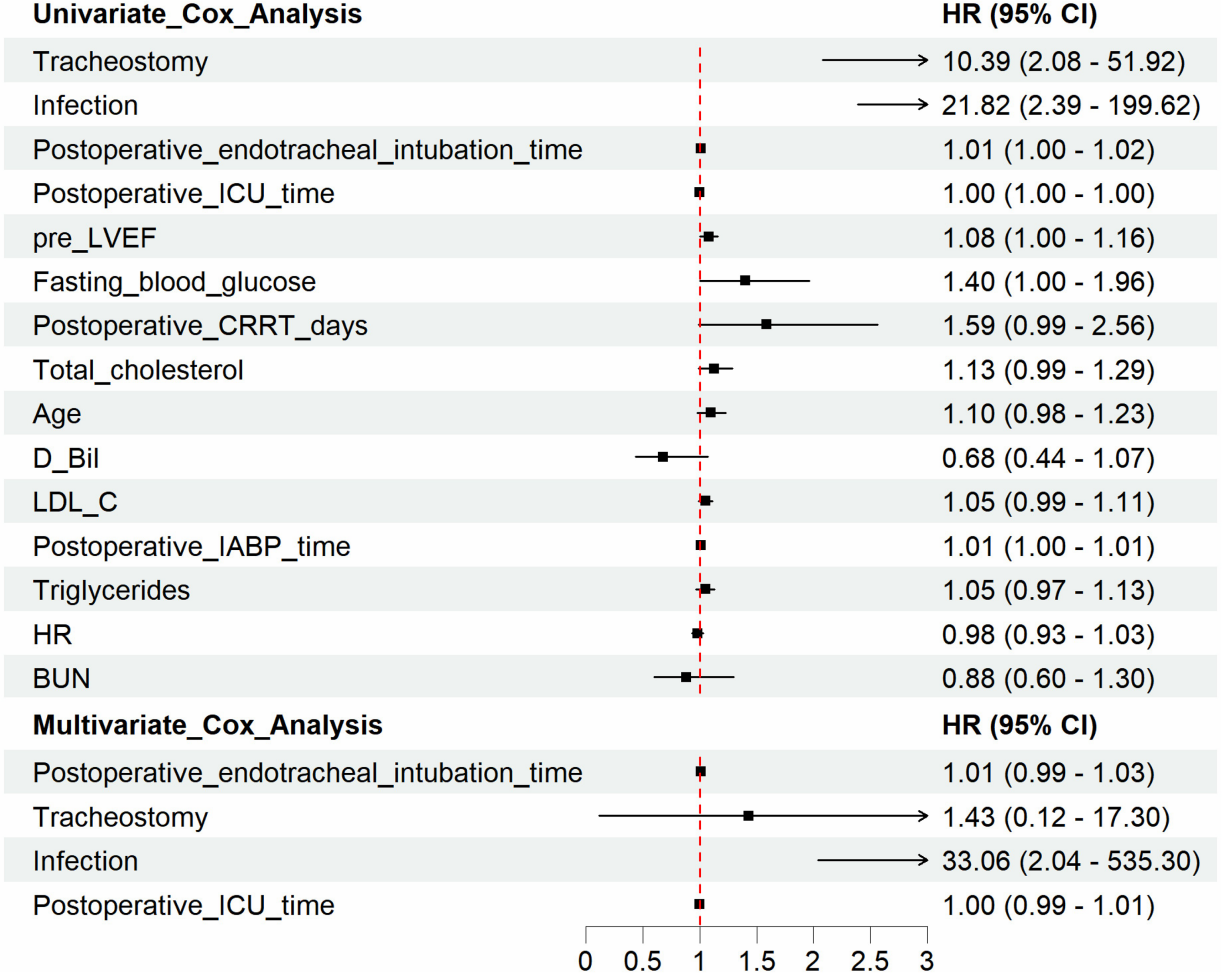
Kaplan–Meier analysis estimates survival in subgroups. Kaplan–Meier analysis estimates survival after surgery with log-rank test, comparing the outcomes in subgroups of valve surgery patients. The results demonstrated that the option of repair or replacement poses no significant difference in postoperative survival.

**Figure 4:**
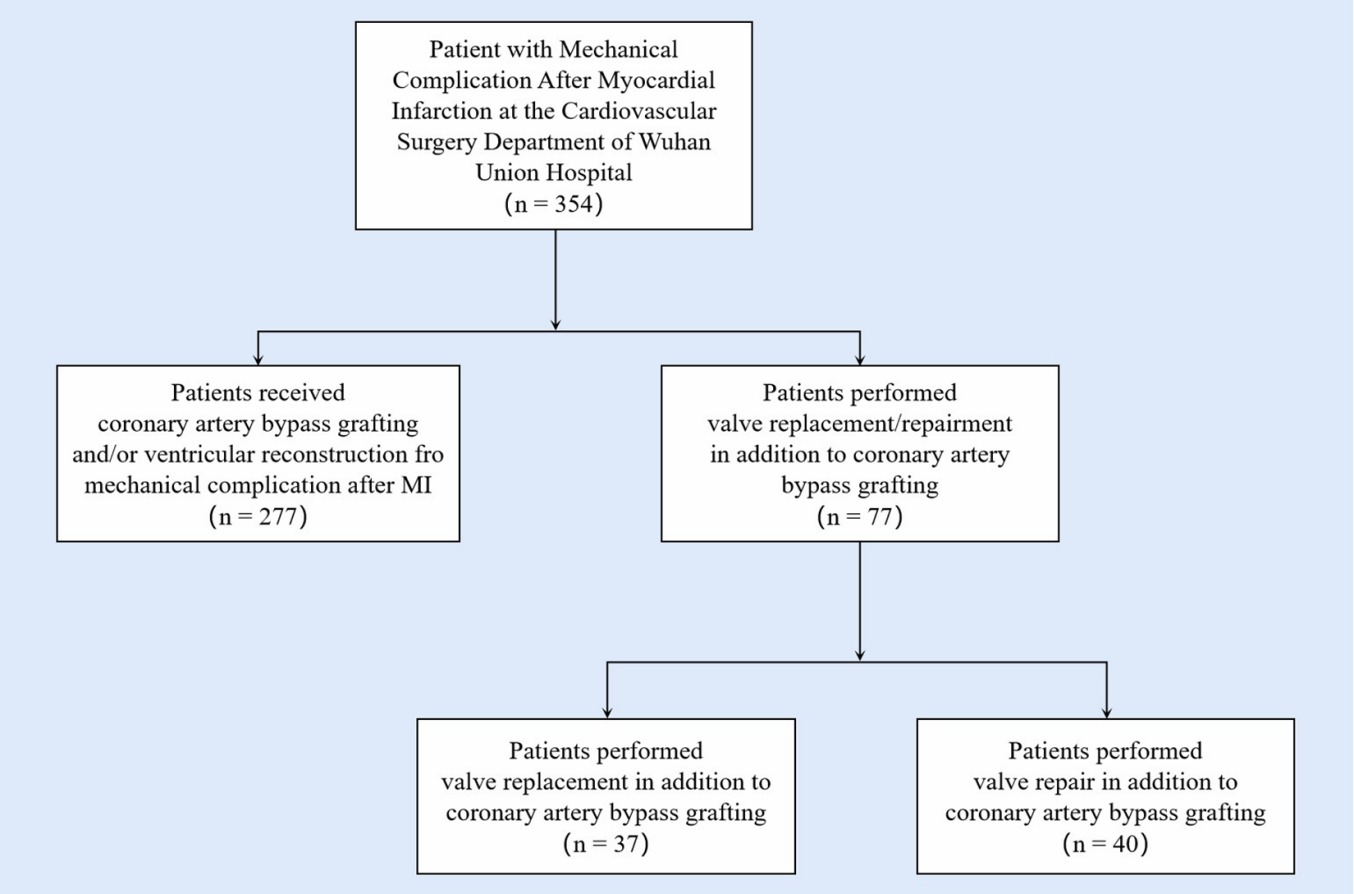
Univariate and multivariate Cox regression analysis identifies risk factors for overall postoperative mortality in subgroups. The univariate and multivariate Cox proportional hazards regression models were performed to identify independent risk factors for patients with valve damage. Univariate analysis showed that the risk factors of mortality were tracheostomy (HR = 10.39, 95% CI, 2.08–51.92, *p* < 0.004), infection (HR = 21.82, 95% CI, 2.39–199.62, *p* = 0.006), postoperative endotracheal intubation time (HR = 1.01, 95% CI, 1.00–1.02, *p* = 0.007), postoperative ICU time (HR = 1.00, 95% CI, 1.00–1.00, *p* = 0.034), pre LVEF (HR = 1.08, 95% CI, 1.00–1.16, *p* = 0.047). Multivariate analysis showed that infection (HR = 33.06, 95% CI, 2.04–535.3, *p* = 0.0138), was an independent and significant predictor of worse outcomes after surgery.

Figure 3 represented the survival curve of the valve surgery subgroup resulting from the Kaplan–Meier survival analysis. No significant disparity in survival probability was shown between replacement and repair (*p* = 0.44). The univariate and multivariate Cox proportional hazards regression models were performed to identify independent risk factors for patients who received concomitant valve surgery. The results were presented in Figure 4. Univariate analysis showed that the risk factors of mortality were tracheostomy (HR = 10.39, 95% CI, 2.08–51.92, *p* < 0.004), infection (HR = 21.82, 95% CI, 2.39–199.62, *p* = 0.006), postoperative endotracheal intubation time (HR = 1.01, 95% CI, 1.00–1.02, *p* = 0.007), postoperative ICU time (HR = 1.00, 95% CI, 1.00–1.00, *p* = 0.034), and pre-LVEF (HR = 1.08, 95% CI, 1.00–1.16, *p* = 0.047). Multivariate analysis showed that infection (HR = 33.06, 95% CI, 2.04–535.3, *p* = 0.0138) was an independent and significant predictor of worse outcomes after surgery. Postoperative endotracheal intubation time (HR = 1.01, 95% CI, 0.99–1.03, *p* < 0.1608), tracheostomy (HR = 1.43, 95% CI, 0.12–17.3, *p* = 0.7764), postoperative ICU time (HR = 1.00, 95% CI, 0.99–1.01, *p* = 0.9068) failed to reach the statistical significance of independent risk factor.

## Discussion

In this study, we retrospectively evaluated the clinical characteristics and prognoses of the long-term outcomes of 354 patients who developed ventricle aneurysms after myocardial infarction and determined the possible influence of concomitant valve surgery. Patients receiving concomitant valve surgery exhibited a higher rate of mortality compared to patients receiving no valve surgery to CABG for ventricle aneurysms. In subgroup analysis, the options of repair or replacement of valve surgery displayed no significant difference in the prognosis outcomes.

Acute myocardial infarction is the most common etiology of ventricular aneurysms, involving the anterior or apical walls of the left ventricle for the highest frequency (13, 14). The structure of the aneurysm is made up of a thin, scarred, or fibrotic myocardial wall, which brings about the characteristics of systolic dysfunction and developing dilation under pressure. In most conditions, the treatments for aneurysms are conservative as the result of restriction of medical conditions and difficulties in early identification (15). Previous studies indicated that revascularization therapy contributes to the improvement of ventricular motion, reverse remodeling, and correcting ventricular dysfunction caused by reversible ischemia. Aneurysmectomy during CABG was suggested as a class IIa recommendation (Level of Evidence: B) under the condition of intractable ventricular arrhythmia and heart failure unresponsive to conventional therapies, by 2004 American College of Cardiology/AHA guidelines on STEMI (16).

Ischemic valve regurgitation also has severe complications following AMI. The incentives for valvular regurgitation are various while papillary muscle ischemia and ventricular remodeling take major places. Papillary muscle ischemia is a rare post-infarction complication with a following rupture rate of less than 0.5% following AMI (17, 18). Given the fact that blood supply differs in papillary muscles, the frequency of rupture is various, depending on the coronary arteries that AMI involves. Posteromedial papillary muscle ruptures 6–12 times more frequently than the anterolateral one, which obtained a dual blood supply from the left anterior descending and circumflex arteries (19–21). Despite the rare prevalence, acute papillary muscle rupture could lead to catastrophic hemodynamics, heart failure, and even cardiogenic shock, with the mortality rate reaching up to 26% and associated with morbidities (22–24). Aside from the acute injury, chronic ischemia also leads to displacement and distortion of papillary muscles, which end in progressive regurgitation and hemodynamic changes. Ventricular remodeling is a chronic complication leading to the formation of aneurysms. Although some studies indicated that ventricular remodeling also makes positive contributions, it still brings impairment to the ejection fraction of the left ventricle. Moreover, it can also lead to the dislocation of the papillary muscle and chordae tendineae, which ends in atrioventricular valve regurgitation. Ventricular reconstruction is considered one of the most effective therapies aiming to prevent exacerbation of valve regurgitation and improve left ventricular function. In this study, all patients underwent CABG, and the majority of patients underwent ventricular reconstruction in addition to CABG (74.1%), which was consistent with the guideline (class IIa, level C) (25).

Several previous studies attempted to compare the results of ventricular reconstruction or CABG for the surgical treatment of ventricular aneurysms with those combined with mitral valve surgery. Nevertheless, the results varied in different studies, and controversial conclusions were drawn among cohorts. In 2008, Sarpity et al. confirmed that the left ventricular reconstruction plus mitral valve surgery group had a higher operative mortality than those who received left ventricular reconstruction alone. However, Zheng et al. found that additional mitral valve surgery did not bring a further probability of mortality to patients who underwent ventricular reconstruction in 2019. One study examined the outcomes in patients with moderate ischemic mitral regurgitation undergoing CABG and demonstrated that the performance of additional restrictive annuloplasty brought no difference in long-term mortality. However, CABG patients with congestive heart failure seemed to benefit from mitral repair (26). Due to the differences in location and size of infarction, studies suffered from the different levels of heterogeneity of patients in cohorts, which weakened the conclusions and required more clinical investigations to confirm the results.

In the present study, patients with ventricular aneurysms who underwent concomitant valve surgery had statistically significantly lower survival probability after surgical treatments. They had significantly greater diameters of cardiac chambers, worse conditions of hypertension, a longer total mechanical ventilation time after surgery. Our findings confirmed that the performance of concomitant valve surgery was significantly associated with lower survival probability and worse postoperative prognosis in the surgical treatment of aneurysms. The results included not only the indications reported in previous studies, but also highlighted the fact that patients with higher total cholesterol levels, higher NYHA classification levels, higher triglyceride levels, and older ages should be given more attention and carefully evaluated before undergoing surgical therapy for post-infarction aneurysms (27, 28). Moreover, the more complex ventricular function is usually associated with the longer ICU stay time, which may lead to the longer intubation and higher risk of infection. In the present study, patients who underwent concomitant valve surgery had longer ICU stays and mechanical ventilation times. Therefore, proactive anti-infective therapy after simultaneous valve surgery has positive implications for better postoperative outcomes.

Surgical intervention is recommended for severe valvular stenosis or regurgitation according to the updated guidelines (29). Nonetheless, this option is preferred under different pre-operative conditions. A randomized controlled trial in 2016 proposed that valve replacement gained a lower probability of postoperative regurgitation and adverse events (30), while some other studies recommend valve repair as a safer and more effective approach for valvular stenosis. Seeing the relatively low incidence of the combined occurrence of valve regurgitation and aneurysm, the choice of repair versus replacement under this condition is still few reported and the risk factors of mortality after surgery are also unexplored. In this study, a total of 77 patients with valve damage received surgical treatment. The result obtained no significant distinction between repair and replacement in survival and other prognostic variables. This was consistent with the conclusion of Russo et al.'s work, which proposed that the option of repair or replacement may not affect the outcomes after surgery (6). Our results extend the study by Castelvecchio et al. We believe that mitral valve repair when necessary may have a positive effect on patient prognosis, but it is still a challenging task (31). Multivariate Cox regression analysis indicated that the postoperative infection is an independent risk factor for mortality after surgery. This alerts that more attention deserved to be given to the sign of infection after surgical treatment in aneurysm patients who underwent concomitant valve surgery. Strict adherence to the principles of proper antibiotic administration, thorough irrigation of the surgical area and negative pressure wound aspiration for patients undergoing concomitant valve surgery may help to reduce the incidence of postoperative adverse events.

Our study expanded on the previous findings of ventricular aneurysm therapy after AMI through a long-term follow-up. The results of our study suggest that patients who underwent concomitant valve surgery had a higher risk of death; however, this difference was not demonstrated by the choice of different valve surgery options (repair or replacement). Pre-operative assessment of the patient's valve status is of significant value in predicting the postoperative status. The options of procedure can be somewhat liberalized according to the actual development of the lesion, without concern for whether the development of a ventricular aneurysm may have a biasing effect on the choice. These findings would provide more information to distinguish patients with higher risks of worse outcomes before the surgery and benefit the postoperative care for patients to obtain better results. Nonetheless, this study still suffered the limitation of sample size and retrospective investigation. A better solution for valve surgical treatment should be raised and further confirmation studies should be conducted in future studies.

## Limitations

There were several limitations in the present study. First, this was a single-center, retrospective and small-sample study and it might suffer from selection bias and the baseline variation may weaken the validity of conclusions; thus, studies with larger sample sizes are required to verify our conclusions. Second, potential confounding could still exist, and the causal relationship between risk factors and outcomes needs to be confirmed in controlled and prospective studies.

## Data Availability

The raw data supporting the conclusions of this article will be made available by the authors, without undue reservation.

## Supplementary material

Supplementary Figure 1

Kaplan–Meier analysis estimates survival of patients with different locations of ventricular aneurysm occurrence. Kaplan–Meier analysis estimates survival after surgery with log-rank test, comparing the outcomes in patients with different locations of ventricular aneurysm occurrence. The results demonstrated that the locations of ventricular aneurysm took place poses no significant difference in postoperative survival.
