# Supplementary material for: Concomitant valve surgery is associated with worse outcomes in surgical treatments of post-infarction ventricular aneurysm
Source: Front Cardiovasc Med. 2023 Aug 15;10:1194374. doi: 10.3389/fcvm.2023.1194374 (PMC10465797; doi:10.3389/fcvm.2023.1194374)
Supplement: Supplementary file 1 [file Table1.docx]

|  | Overall | No Valve Surgery | Valve Surgery | p |
| --- | --- | --- | --- | --- |
| n | 354 | 277 | 77 |  |
| **Clinical Characteristics** |  |  |  |  |
| Gender (%) |  |  |  | 0.954 |
| male | 290 (81.9) | 227 (81.9) | 63 (81.8) |  |
| female | 64 (18.1) | 50 (18.1) | 14 (18.2) |  |
| Age (year) | 57.80 (8.08) | 57.67 (8.22) | 58.29 (7.60) | 0.554 |
| BMI (kg/m^2^) | 24.58 (3.38) | 24.39 (3.50) | 25.27 (2.86) | 0.042 |
| Hypertension (%) |  |  |  | <0.001 |
| I | 50 (14.1) | 32 (11.6) | 18 (23.4) |  |
| II | 40 (11.3) | 24 (8.7) | 16 (20.8) |  |
| III | 102 (28.8) | 79 (28.5) | 23 (29.9) |  |
| NYHA level (%) |  |  |  | <0.001 |
| 1 | 64 (18.1) | 50 (18.1) | 14 (18.2) |  |
| 2 | 130 (36.7) | 115 (41.5) | 15 (19.5) |  |
| 3 | 124 (35.1) | 84 (30.3) | 40 (52.0) |  |
| 4 | 36 (10.2) | 28 (10.1) | 8 (10.4) |  |
| Left Heart Enlargement (%) | 240 (67.8) | 181 (65.3) | 59 (76.6) | 0.073 |
| HR (bpm) | 77.49 (17.23) | 78.08 (17.74) | 75.35 (15.14) | 0.219 |
| **Echocardiography Examinations** |  |  |  |  |
| AAO (cm) | 3.38 (0.36) | 3.37 (0.38) | 3.41 (0.29) | 0.418 |
| LA (cm) | 4.13 (0.57) | 4.05 (0.52) | 4.41 (0.62) | <0.001 |
| LV (cm) | 5.79 (0.73) | 5.70 (0.72) | 6.11 (0.71) | <0.001 |
| IVS (cm) | 0.94 (0.21) | 0.93 (0.22) | 1.00 (0.15) | 0.007 |
| RA (cm) | 3.75 (0.63) | 3.71 (0.66) | 3.88 (0.51) | 0.037 |
| RV (cm) | 3.57 (0.51) | 3.53 (0.48) | 3.70 (0.58) | 0.008 |
| PA (cm) | 2.52 (0.38) | 2.50 (0.38) | 2.60 (0.37) | 0.040 |
| LVEF (%) | 44.85 (25.01) | 44.70 (27.78) | 45.41 (10.06) | 0.824 |
| LV End-Diastolic Volume (mL) | 227.07 (76.98) | 224.87 (77.15) | 235.10 (76.86) | 0.470 |
| LV End-Systolic Volume (mL) | 157.08 (64.37) | 160.50 (66.41) | 144.56 (55.30) | 0.177 |
| Mean mitral regurgitation of group (m/s) | 0.22 (1.01) | 0.25 (1.06) | 0.14 (0.81) | 0.567 |
| **Blood Biomarkers** |  |  |  |  |
| T Bil (mg/dl) | 13.43 (8.19) | 13.66 (8.66) | 12.60 (6.22) | 0.314 |
| D Bil (mg/dl) | 4.91 (3.72) | 5.05 (3.87) | 4.39 (3.05) | 0.17 |
| ALT (unit) | 30.03 (21.94) | 30.46 (23.28) | 28.48 (16.29) | 0.485 |
| AST (unit) | 27.66 (22.40) | 28.47 (24.35) | 24.74 (12.89) | 0.197 |
| BUN (mmol/L) | 6.67 (2.97) | 6.66 (3.11) | 6.70 (2.42) | 0.924 |
| SCr (umol/L) | 92.89 (60.58) | 92.93 (66.85) | 92.77 (28.52) | 0.984 |
| UA (umol/L) | 381.65 (124.36) | 374.42 (125.79) | 407.66 (116.18) | 0.038 |
| Total cholesterol (mg/dl) | 4.02 (1.84) | 3.94 (1.48) | 4.31 (2.75) | 0.111 |
| Triglycerides (mmol/L) | 2.14 (2.77) | 2.09 (1.82) | 2.33 (4.85) | 0.491 |
| HDL-C (mmol/L) | 1.40 (1.36) | 1.35 (1.14) | 1.61 (1.95) | 0.14 |
| LDL-C (mmol/L) | 3.57 (4.45) | 3.43 (3.89) | 4.05 (6.06) | 0.284 |
| **Coronary artery lesion** |  |  |  |  |
| LAD (%) | 178 (50.3) | 140 (50.5) | 38 (49.4) | 0.898 |
| LCX (%) | 168 (47.5) | 136 (49.1) | 32 (41.6) | 0.249 |
| RCA (%) | 168 (47.5) | 132 (47.7) | 36 (46.8) | 0.898 |
| PDA (%) | 78 (22.0) | 58 (20.9) | 20 (26.0) | 0.354 |

**Table 1. Baseline characteristics of study participants.**

BMI, Body Mass Index; NYHA, New York Heart Association; HR, Heart Rate; AAO, Ascending Aorta; LA, Left Atrium; LV, Left Ventricular; IVS, Ventricular Septum; RA, Right Atrium; RV, Right Ventricular; PA, Pulmonary Artery; LVEF, Left Ventricular Ejection Fraction; T Bil, Total bilirubin; D Bil, Direct Bilirubin; ALT, Alanine Transaminase; AST, Aspartate Transaminase; BUN, blood urea nitrogen; SCr, Serum Creatinine; UA, Uric Acid; HDL-C, High-Density Lipoprotein Cholesterol; LDL-C, Low-Density Lipoprotein Cholesterol; LAD, Left Anterior Descending; LCX, Left Circumflex Artery; RCA, Right Coronary Artery; PDA, Posterior Descending Artery.
