# Supplementary material for: Concomitant valve surgery is associated with worse outcomes in surgical treatments of post-infarction ventricular aneurysm
Source: Front Cardiovasc Med. 2023 Aug 15;10:1194374. doi: 10.3389/fcvm.2023.1194374 (PMC10465797; doi:10.3389/fcvm.2023.1194374)
Supplement: Supplementary file 2 [file Table2.docx]

|  | Overall | No Valve Surgery | Valve Surgery | p |
| --- | --- | --- | --- | --- |
| n | 354 | 277 | 77 |  |
| Tracheostomy (%) | 14 (4.0) | 8 (2.9) | 6 (7.8) | 0.089 |
| Patients underwent ventricular reconstruction (%) | 190 (53.6) | 178 (64.3) | 12 (15.6) | <0.001 |
| Postopreative IABP (%) | 130 (36.7) | 98 (35.4) | 32 (41.6) | 0.350 |
| Respiratory complications (%) | 58 (16.4) | 40 (14.4) | 18 (23.4) | 0.080 |
| Renal insufficiency (%) | 8 (2.3) | 6 (2.2) | 2 (2.6) | 0.686 |
| Infection (%) | 56 (15.8) | 40 (14.4) | 16 (20.8) | 0.216 |
| Sputum culture positive (%) | 54 (15.3) | 38 (13.7) | 16 (20.8) | 0.151 |
| rebridge (%) | 4 (1.1) | 2 (0.7) | 2 (2.6) | 0.207 |
| ACS (%) | 14 (4.0) | 8 (2.9) | 6 (7.8) | 0.089 |
| Postoperative ICU time  (hour) | 153.95 (114.86) | 147.18 (101.50) | 178.34 (151.98) | 0.035 |
| Postoperative endotracheal intubation time (hour) | 60.92 (47.23) | 58.00 (44.18) | 71.40 (55.97) | 0.027 |
| Postoperative IABP time  (hour) | 58.15 (89.40) | 55.88 (87.09) | 66.31 (97.41) | 0.366 |
| Postoperative hospital stay (day) | 19.15 (9.06) | 19.04 (9.16) | 19.57 (8.71) | 0.647 |
| Total mechanical ventilation time (hour) | 66.63 (80.60) | 60.20 (46.17) | 89.77 (147.44) | 0.004 |
| One-year LVEF (%) | 43.05 (11.34) | 43.31 (11.91) | 41.44 (7.18) | 0.651 |
| All-cause mortality (%) | 26 (7.34) | 16 (5.78) | 10 (13.0) | 0.032 |

**Table 2. Prognostic information for patients undergoing surgery for post-infarction ventricular aneurysm.**

IABP, Intra-Aortic Balloon Pump; ACS, Acute Coronary Syndrome; ICU, Intensive Care Unit; LVEF, Left Ventricular Ejection Fraction.
