# Supplementary material for: Concomitant valve surgery is associated with worse outcomes in surgical treatments of post-infarction ventricular aneurysm
Source: Front Cardiovasc Med. 2023 Aug 15;10:1194374. doi: 10.3389/fcvm.2023.1194374 (PMC10465797; doi:10.3389/fcvm.2023.1194374)
Supplement: Supplementary file 3 [file Table3.docx]

| Univariate Cox Analysis | | Hazard Ratio | p | |  |
| --- | --- | --- | --- | --- | --- |
| Total cholesterol | | 1.22 (1.08 - 1.37) | <0.01 | |  |
| LDL-C | | 1.08 (1.02 - 1.14) | 0.01 | |  |
| Age | | 1.09 (1.02 - 1.16) | 0.01 | |  |
| Triglycerides | | 1.09 (1.01 - 1.17) | 0.02 | |  |
| NYHA level | | 1.88 (1.06 - 3.36) | 0.03 | |  |
| Valve damage | | 2.93 (1.09 - 7.83) | 0.03 | |  |
| ALT | | 1.01 (1.00 - 1.03) | 0.14 | |  |
| Operation history | | 0.52 (0.17 - 1.57) | 0.24 | |  |
| Hypertension | | 1.22 (0.87 - 1.71) | 0.25 | |  |
| D-Bil | | 0.93 (0.78 - 1.12) | 0.45 | |  |
| BMI | | 0.96 (0.83 - 1.11) | 0.55 | |  |
| HDL-C | | 1.08 (0.81 - 1.43) | 0.60 | |  |
| BUN | | 1.03 (0.93 - 1.14) | 0.62 | |  |
| AST | | 1.00 (0.97 - 1.02) | 0.67 | |  |
| Left heart enlargement | | 0.89 (0.35 - 2.27) | 0.82 | |  |
| T-Bil | | 0.99 (0.93 - 1.06) | 0.84 | |  |
| Gender | | 0.89 (0.26 - 3.06) | 0.85 | |  |
| SCr | | 1.00 (1.00 - 1.01) | 0.92 | |  |
| HR | | 1.00 (0.97 - 1.03) | 0.98 | |  |
| Multivariate Cox Analysis | Hazard Ratio | | | p | |
| Total cholesterol | 1.68 (1.45 - 1.94) | | | ＜0.001 | |
| Postopreative IABP | 6.29 (2.05 - 19.3) | | | 0.0013 | |
| NYHA level | 2.84 (1.50 - 5.41) | | | 0.0014 | |
| Triglycerides | 1.09 (1.01 - 1.17) | | | 0.0283 | |
| Age | 1.02 (0.95 - 1.09) | | | 0.5623 | |

**Table 3. Results of univariate and multivariate Cox analysis for the prognosis after surgery.**

LDL-C, Low-Density Lipoprotein Cholesterol; ALT, Alanine Transaminase; D Bil, Direct Bilirubin; BMI, Body Mass Index; HDL-C, High-Density Lipoprotein Cholesterol; BUN, blood urea nitrogen; T Bil, Total bilirubin; SCr, Serum Creatinine; HR, Heart Rate; IABP, Intra-Aortic Balloon Pump; NYHA, New York Heart Association.
