# Supplementary material for: Concomitant valve surgery is associated with worse outcomes in surgical treatments of post-infarction ventricular aneurysm
Source: Front Cardiovasc Med. 2023 Aug 15;10:1194374. doi: 10.3389/fcvm.2023.1194374 (PMC10465797; doi:10.3389/fcvm.2023.1194374)
Supplement: Supplementary file 4 [file Table4.docx]

|  | Overall | Repair | Replacement | p |
| --- | --- | --- | --- | --- |
| n | 77 | 40 | 37 |  |
| **Clinical Characteristics** |  |  |  |  |
| Gender (%) |  |  |  |  |
| male | 63 (81.8) | 30 (75.0) | 33 (89.2) | 0.143 |
| female | 14 (18.2) | 10 (25.0) | 4 (10.8) |  |
| Age (year) | 58.29 (7.60) | 59.90 (6.97) | 56.54 (7.95) | 0.052 |
| BMI (kg/m^2^) | 25.27 (2.86) | 25.33 (2.82) | 25.20 (2.94) | 0.837 |
| HR (bpm) | 75.35 (15.14) | 75.85 (13.85) | 74.81 (16.60) | 0.766 |
| **Echocardiography Examinations** |  |  |  |  |
| AAO (cm) | 3.41 (0.29) | 3.34 (0.33) | 3.48 (0.24) | 0.047 |
| LA (cm) | 4.41 (0.62) | 4.45 (0.56) | 4.36 (0.69) | 0.528 |
| LV (cm) | 6.11 (0.71) | 6.24 (0.71) | 5.97 (0.68) | 0.085 |
| IVS (cm) | 1.00 (0.15) | 0.93 (0.14) | 1.08 (0.12) | <0.001 |
| RA (cm) | 3.88 (0.51) | 3.86 (0.53) | 3.91 (0.49) | 0.698 |
| RV (cm) | 3.70 (0.58) | 3.70 (0.61) | 3.70 (0.56) | 0.97 |
| PA (cm) | 2.60 (0.37) | 2.68 (0.36) | 2.52 (0.36) | 0.053 |
| LVEF (%) | 45.41 (10.06) | 45.07 (10.56) | 45.79 (9.63) | 0.757 |
| **Blood Biomarkers** |  |  |  |  |
| T Bil (mg/dl) | 12.60 (6.22) | 13.39 (6.64) | 11.74 (5.69) | 0.246 |
| D Bil (mg/dl) | 4.39 (3.05) | 5.18 (3.78) | 3.54 (1.66) | 0.017 |
| ALT (unit) | 28.48 (16.29) | 28.60 (15.29) | 28.35 (17.53) | 0.947 |
| AST (unit) | 24.74 (12.89) | 27.90 (15.90) | 21.32 (7.33) | 0.024 |
| BUN (mmol/L) | 6.70 (2.42) | 6.71 (1.89) | 6.68 (2.92) | 0.964 |
| SCr (umol/L) | 92.77 (28.52) | 88.09 (20.78) | 97.82 (34.62) | 0.135 |
| UA (umol/L) | 407.66 (116.18) | 421.53 (137.53) | 392.66 (86.96) | 0.279 |
| Total cholesterol (mg/dl) | 4.31 (2.75) | 4.41 (3.37) | 4.21 (1.91) | 0.763 |
| Triglycerides (mmol/L) | 2.33 (4.85) | 2.98 (6.61) | 1.64 (1.16) | 0.23 |
| HDL-C (mmol/L) | 1.61 (1.95) | 1.98 (2.53) | 1.21 (0.90) | 0.084 |
| LDL-C (mmol/L) | 4.05 (6.06) | 5.23 (7.76) | 2.78 (3.03) | 0.076 |
| **Prognosis Information** |  |  |  |  |
| Tracheostomy (%) | 6 (7.8) | 4 (10.0) | 2 (5.4) | 0.546 |
| Postopreative IABP (%) | 32 (41.6) | 20 (50.0) | 12 (32.4) | 0.165 |
| Respiratory complications (%) | 18 (23.4) | 6 (15.0) | 12 (32.4) | 0.106 |
| Renal insufficiency (%) | 2 (2.6) | 2 (5.0) | 0 (0.0) | 0.494 |
| Infection (%) | 16 (20.8) | 6 (15.0) | 10 (27.0) | 0.263 |
| Sputum culture positive (%) | 16 (20.8) | 6 (15.0) | 10 (27.0) | 0.263 |
| Rebridge (%) | 2 (2.6) | 2 (5.0) | 0 (0.0) | 0.494 |
| Postoperative ICU time  (hour) | 177.14 (152.99) | 186.80 (184.15) | 166.70 (111.63) | 0.568 |
| Postoperative endotracheal intubation time (hour) | 70.84 (56.45) | 76.60 (66.71) | 64.61 (42.76) | 0.355 |
| Postoperative IABP time  (hour) | 66.91 (97.14) | 66.85 (89.67) | 66.97 (105.87) | 0.996 |
| Postoperative hospital stay (day) | 19.38 (8.98) | 20.55 (9.72) | 18.11 (8.05) | 0.236 |
| Total mechanical ventilation time (hour) | 89.61 (147.50) | 111.40 (199.28) | 66.05 (42.50) | 0.179 |

**Table 4. Subgroup analysis of patients receiving concomitant valve surgery.**

BMI, Body Mass Index; HR, Heart Rate; AAO, Ascending Aorta; LA, Left Atrium; LV, Left Ventricular; IVS, Ventricular Septum; RA, Right Atrium; RV, Right Ventricular; PA, Pulmonary Artery; LVEF, Left Ventricular Ejection Fraction; T Bil, Total bilirubin; D Bil, Direct Bilirubin; ALT, Alanine Transaminase; AST, Aspartate Transaminase; BUN, blood urea nitrogen; SCr, Serum Creatinine; UA, Uric Acid; HDL-C, High-Density Lipoprotein Cholesterol; LDL-C, Low-Density Lipoprotein Cholesterol; IABP, Intra-Aortic Balloon Pump; ACS, Acute Coronary Syndrome; ICU, Intensive Care Unit; LVEF, Left Ventricular Ejection Fraction.
