# Supplementary figures and images for: Concomitant valve surgery is associated with worse outcomes in surgical treatments of post-infarction ventricular aneurysm
Source: Front Cardiovasc Med. 2023 Aug 15;10:1194374. doi: 10.3389/fcvm.2023.1194374 (PMC10465797; doi:10.3389/fcvm.2023.1194374)

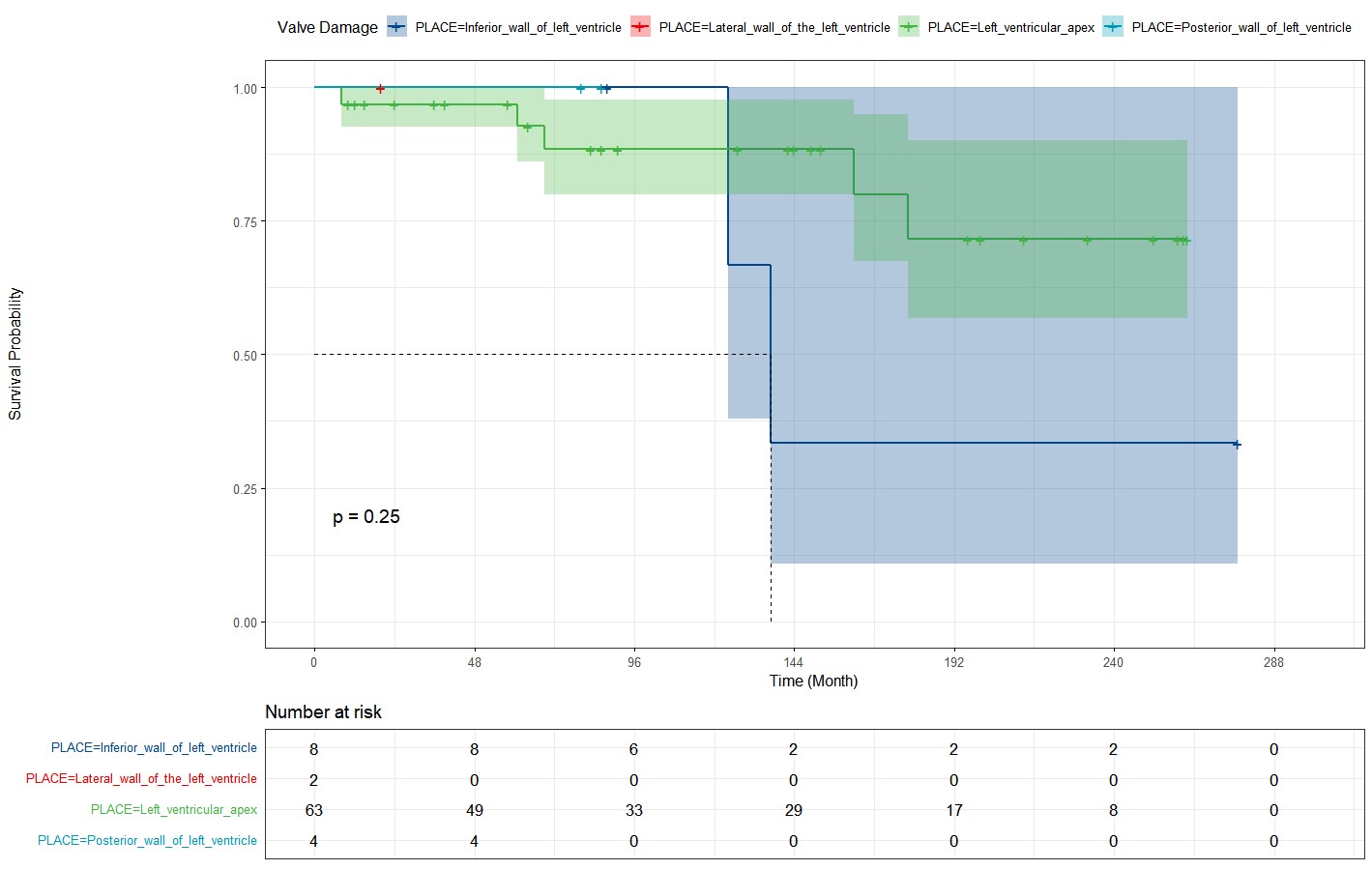

Supplement: Supplementary file 5 [file Image1.jpeg]
